# Evaluation of the relationship between insulin resistance and nicotine dependence: A cross-sectional study

**DOI:** 10.18332/tid/216382

**Published:** 2026-02-19

**Authors:** Selma Saruhan, Mehmet Kocabaş, Gamzenur Teker Tekbil, Yılmaz Sezgin

**Affiliations:** 1 Department of Family Medicine, Kanuni Training and Research Hospital, University of Health Sciences, Trabzon, Türkiye; 2 Department of Family Medicine, Yavuz Selim Bone Diseases and Rehabilitation Hospital, Trabzon, Türkiye

**Keywords:** insulin resistance, triglycerides, nicotine dependence, tobacco use disorder, blood glucose

## Abstract

**INTRODUCTION:**

The mechanisms underlying the association between insulin resistance and nicotine dependence remain incompletely understood. This study aimed to examine the association between insulin resistance, assessed by the triglyceride–glucose (TyG) index, and nicotine dependence, measured by the Fagerström test for nicotine dependence (FTND), among adult exclusive combustible cigarette smokers.

**METHODS:**

This cross-sectional study was conducted between February and June 2025 in a tertiary care setting and included 169 adult exclusive combustible cigarette smokers aged 18–80 years. Individuals with diabetes mellitus or those receiving lipid-lowering therapy were excluded. Nicotine dependence was assessed using the Fagerström test for nicotine dependence (FTND), and insulin resistance was estimated using the triglyceride–glucose (TyG) index. Correlation analyses and multivariable linear regression were performed to examine factors associated with the TyG index.

**RESULTS:**

The mean age of the participants was 40.23 ± 12.95 years, and 55.6% were male. The mean TyG index was 8.51 ± 0.56, and the mean FTND score was 4.86 ± 2.32. The TyG index showed positive correlations with FTND score (r=0.280; p<0.001), age (r=0.261; p=0.001), and pack-years (r=0.218; p=0.004), and was significantly higher in males than in females (p<0.001). In multivariable linear regression analysis, older age, male sex, and higher FTND scores were independently associated with higher TyG index values.

**CONCLUSIONS:**

The findings suggest a potential association between nicotine dependence and insulin resistance among adult exclusive combustible cigarette smokers. Prospective studies are needed to clarify the direction and clinical significance of this association.

## INTRODUCTION

Smoking is one of the leading preventable causes of death worldwide and is associated with nearly eight million deaths each year^1^. Tobacco smoke contains more than 7000 chemicals, including nicotine, carbon monoxide, and heavy metals^2^. Nicotine binds to nicotinic acetylcholine receptors in the central nervous system, stimulating dopaminergic reward pathways and playing a key role in the development of addiction^3^. The Fagerström test for nicotine dependence (FTND) is a widely used and validated scale in both clinical and epidemiological studies for assessing the level of nicotine dependence^4^.

Insulin resistance is a condition characterized by a reduced biological response to insulin and plays an important role in the development of type 2 diabetes, metabolic syndrome, and cardiovascular diseases^5^. The triglyceride–glucose (TyG) index, calculated using fasting triglyceride and glucose levels, is a practical and reliable marker associated with insulin resistance^6^. In recent years, evidence has suggested that alterations in brain insulin signaling may be related to reward mechanisms through dopaminergic pathways, potentially associated with addictive behaviors. Therefore, new hypotheses have emerged proposing that insulin resistance, similar to nicotine dependence, may also influence addictive behaviors through dopaminergic pathways^7,8^.

Studies examining the relationship between smoking and insulin resistance generally emphasize that smoking negatively affects insulin sensitivity^9-11^. Consistent with this perspective, research on the role of insulin signaling in the dopaminergic system suggests that this relationship may involve shared neural pathways rather than a direct causal link^7,8^. Therefore, this study aimed to investigate the association between nicotine dependence, assessed by the FTND, and insulin resistance, evaluated using the TyG index, among adult exclusive cigarette smokers.

## METHODS

### Study design and sample selection

This cross-sectional study was conducted between February and June 2025 at the Smoking Cessation Clinic and the Family Medicine Clinic of Trabzon Kanuni Training and Research Hospital. Ethical approval for the study was obtained from the Scientific Research Ethics Committee of the University of Health Sciences, Trabzon Faculty of Medicine (Approval No: 2025/176; Date: February 4, 2025). Written informed consent was obtained from all participants.

The sample size was calculated using the G*Power 3.1 software^12^ for correlation analysis. Assuming a two-tailed hypothesis, a type I error rate of 5%, an effect size of r=0.50, and a statistical power of 80%, the minimum required sample size was estimated to be 66 participants. The selected effect size corresponds to a large effect according to Cohen’s classification and was chosen to ensure sufficient power in the absence of prior population-specific estimates. As the study protocol predefined that multivariable regression analyses would be performed following significant correlations, a larger sample size was recruited to improve the robustness of the analyses and to allow adjustment for potential confounding variables.

**Figure 1:**
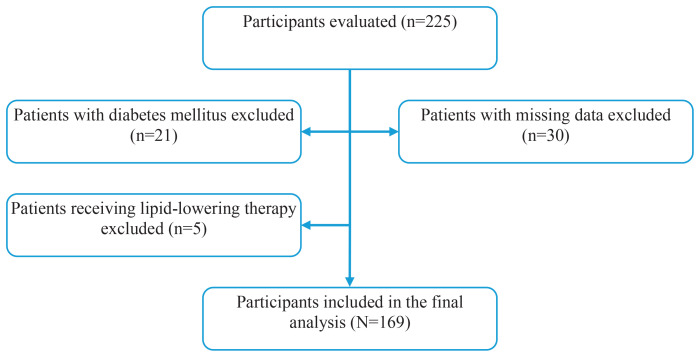
Flow diagram of participant selection in a cross-sectional study conducted at Trabzon Kanuni Training and Research Hospital, Türkiye, 2025 (N=169)

Participants aged 18–80 years who volunteered to participate and reported exclusive combustible cigarette use were included in the study. Individuals using other nicotine products, including e-cigarettes, heated tobacco products, nicotine pouches, or smokeless tobacco, were excluded. Additional exclusion criteria included a diagnosis of diabetes mellitus (n=21), current use of lipid-lowering therapy (n=5), and missing baseline data (n=30). As a result, 56 individuals were excluded, and 169 participants were included in the final analyses. The participant selection process is illustrated in Figure 1.

### Data collection

The participants’ levels of nicotine dependence were assessed during their outpatient clinic visits using the Fagerström test for nicotine dependence through face-to-face interviews. Demographic information, including age (years) and sex, as well as smoking characteristics such as duration of smoking (years) and the number of cigarettes smoked per day (cigarettes/day), were obtained through structured interviews and verified using outpatient clinic records.

Biochemical data, including fasting glucose (mg/dL) and triglyceride levels (mg/dL), were retrieved from the electronic hospital information system. All laboratory measurements were obtained after an overnight fast and were collected on the same day as the clinical evaluation to ensure temporal consistency. Blood samples were analyzed in the hospital’s central laboratory using standardized automated methods.

### Variables and measurements

The dependent variable of the study was the TyG index, an established surrogate marker of insulin resistance. The TyG index was calculated using fasting glucose and triglyceride measurements according to the formula^6^: TyG = ln ([Triglyceride (mg/dL) × Glucose (mg/dL)]/2).

Independent variables included the FTND score, age, sex, and pack-years.

The FTND consists of six items yielding a total score ranging from 0 to 10, with higher scores indicating greater nicotine dependence^4^. Although the FTND is formally an ordinal scale, it was treated as a continuous variable in the present study to preserve statistical power and avoid loss of information. All biochemical measurements were obtained from fasting blood samples collected in the morning and analyzed in the hospital laboratory using standardized automated methods. Pack-years were calculated by multiplying the duration of smoking (years) by the number of cigarettes smoked per day (cigarettes/day) and dividing by 20, and were treated as a continuous variable. Age was recorded as a continuous variable, and sex was coded as a categorical variable (male, female).

### Statistical analysis

Data analysis was performed using IBM SPSS Statistics version 25.0 (IBM Corp., Armonk, NY, USA)^13^. The normality of continuous variables was evaluated using skewness–kurtosis values (acceptable range: -1.5 to +1.5) and complemented by visual inspection of histograms and Q–Q plots. Both the TyG index and FTND score showed approximately normal distributions. Continuous variables were summarized as mean ± standard deviation, while categorical variables were presented as frequency (n) and percentage (%). Pearson correlation coefficients were used to examine associations between continuous variables, including FTND score, TyG index, age, and pack-years. Interpretation of correlation coefficients followed conventional thresholds: weak (r=0.10–0.29), moderate (r=0.30–0.49), and strong (r ≥0.50).

Differences in continuous variables between male and female participants were assessed using independent-samples t-tests.

Candidate variables for multivariable linear regression were selected based on their clinical relevance and observed associations in univariable analyses. Variables that showed significant correlations with the TyG index, including age and pack-years, as well as sex, based on their clinical relevance and significant differences in TyG levels in univariable comparisons, were included in the multivariable model together with the FTND score to examine the association between nicotine dependence and insulin resistance. The coefficient of determination (R²) was used to assess the proportion of variance in the TyG index explained by the multivariable regression model. Multicollinearity was examined using the variance inflation factor (VIF), and a VIF <5 was considered acceptable. All statistical tests were two-tailed, and a p<0.05 was accepted as the threshold for statistical significance.

## RESULTS

**Figure 2:**
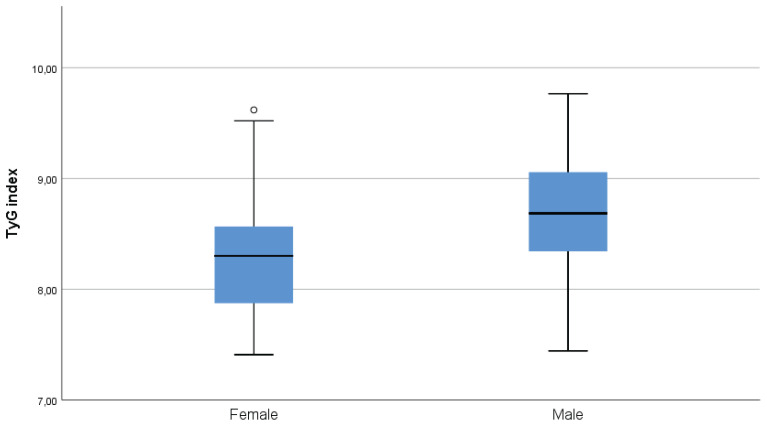
Distribution of the triglyceride–glucose (TyG) index, by sex, among adult exclusive combustible cigarette smokers in a cross-sectional study conducted at Trabzon Kanuni Training and Research Hospital, Türkiye, 2025 (N=169)

A total of 169 adult exclusive combustible cigarette smokers were included in the analysis. The mean age of the participants was 40.2 ± 12.9 years, and 55.6% were male. The mean TyG index was 8.51 ± 0.56, and the mean FTND score was 4.86 ± 2.32 (Table 1). The TyG index showed significant positive correlations with FTND score (r=0.280; p<0.001), age (r=0.261; p=0.001), and pack-years (r=0.218; p=0.004). FTND score was positively correlated with pack-years (r=0.242; p=0.002), while no significant association was observed between FTND score and age (Table 2). The TyG index was significantly higher in males than in females based on an independent-samples t-test (p<0.001) (Figure 2).

**Table 1:** Sociodemographic and biochemical data of adult exclusive combustible cigarette smokers in a cross-sectional study conducted at Trabzon Kanuni Training and Research Hospital, Türkiye, 2025 (N=169)

*Characteristics*	*Mean ± SD*	*Range*
**Age** (years)	40.23 ± 12.95	18–79
**Sex,** n (%)		
Female	75 (44.4)	-
Male	94 (55.6)	
**Health status**		
Triglycerides (mg/dL)	131.3 ± 69.19	40–307
Glucose (mg/dL)	87.16 ± 11.11	62–123
TyG index	8.51 ± 0.56	7.41–9.76
FTND score	4.86 ± 2.32	0–10
Cigarettes smoked per day	21.6 ± 11.86	1–80
Duration of smoking (years)	17.98 ± 11.02	1–50
Pack-years	20.07 ± 17.47	0.4–80

TyG: triglyceride–glucose index. FTND: Fagerström test for nicotine dependence.

**Table 2:** Correlations between TyG index, FTND score, and demographic/smoking-related variables among adult exclusive combustible cigarette smokers in a cross-sectional study conducted at Trabzon Kanuni Training and Research Hospital, Türkiye, 2025 (N=169)

*Variables*	*TyG index*	*FTND score*
**Age** (years)	r=0.261 p=0.001	r=0.025 p=0.751
**Pack-years**	r=0.218 p=0.004	r=0.242 p=0.002
**FTND score**	r=0.280 p<0.001	-

Pearson’s correlation coefficient was used to assess associations between continuous variables. All statistical tests were two-tailed. TyG: triglyceride–glucose index. FTND: Fagerström test for nicotine dependence.

**Figure 3:**
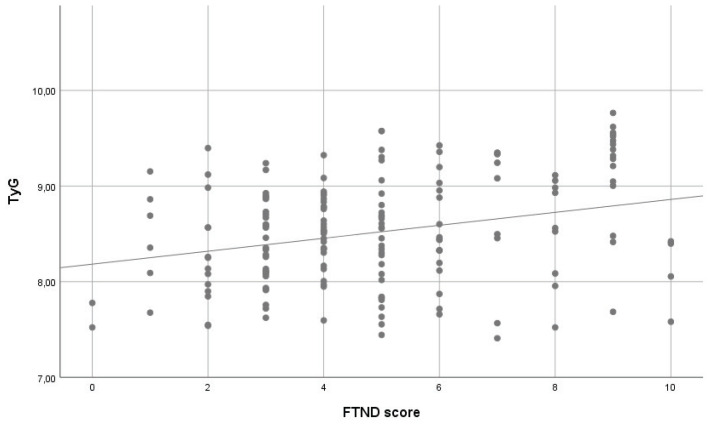
Scatter plot illustrating the association between FTND score and TyG index among adult exclusive combustible cigarette smokers in a cross-sectional study conducted at Trabzon Kanuni Training and Research Hospital, Türkiye, 2025 (N=169)

In multivariable linear regression analysis with the TyG index as the dependent variable, higher FTND scores were independently associated with higher TyG levels. In Model 1, which included FTND score only, a significant association was observed between FTND score and the TyG index (β=0.068, SE=0.018; p<0.001). In Model 2, after adjustment for age, sex, and pack-years, FTND score remained significantly associated with the TyG index (β=0.067, SE=0.017; p<0.001). Age (β=0.011, SE=0.004; p=0.002) and male sex (β=0.354, SE=0.078; p<0.001) were also significantly associated with higher TyG levels, whereas pack-years showed no significant association (p=0.703). No evidence of multicollinearity was observed (all VIF values <5) (Table 3). The association between FTND score and the TyG index is shown in Figure 3.

**Table 3:** Multivariable linear regression analysis of factors associated with the TyG index among adult exclusive combustible cigarette smokers in a cross-sectional study conducted at Trabzon Kanuni Training and Research Hospital, Türkiye, 2025 (N=169)

*Model*	*Variable*	*β*	*SE*	*t*	*p*	*Tolerance*	*VIF*
1	Constant	8.184	0.097	84.675	<0.001	-	-
	FTND score	0.068	0.018	3.772	<0.001	1.000	1.000
2	Constant	7.574	0.159	47.674	<0.001	-	-
	FTND score	0.067	0.017	3.896	<0.001	0.926	1.080
	Age (years)	0.011	0.004	3.098	0.002	0.705	1.418
	Sex (male)	0.354	0.078	4.555	<0.001	0.974	1.027
	Pack-years	-0.001	0.003	-0.382	0.703	0.650	1.538

Model 1: FTND score only. Model 2: FTND score, age, sex, and pack-years included. β: unstandardized regression coefficient. SE: standard error. Model 1: F=14.226, p<0.001; R²=0.078. Model 2: F=12.922, p<0.001; R²=0.221. TyG: triglyceride–glucose index; FTND: Fagerström test for nicotine dependence.

## DISCUSSION

This study is novel as it examined the association between insulin resistance, assessed by the TyG index, and nicotine dependence, measured by the FTND. Our findings showed that higher TyG index values were independently associated with higher FTND scores, as well as with older age and male sex. In addition, the FTND score was positively correlated with both the TyG index and cumulative smoking exposure measured by pack-years. Taken together, these findings indicate that insulin resistance is associated with nicotine dependence beyond smoking intensity alone; however, given the cross-sectional design, no causal or directional inferences can be drawn.

Our findings are consistent with the existing literature. Previous studies have shown that cigarette smoking is associated with reduced insulin sensitivity, impaired glucose metabolism, and an increased risk of type 2 diabetes^14-16^. Studies using the TyG index have also reported significant associations between smoking status and TyG levels^16,17^. The positive correlation observed in our study between FTND score and the TyG index is in line with these reports. However, human studies directly examining the association between nicotine dependence and insulin resistance remain limited. In this context, our results are consistent with experimental findings by Richardson et al.^8^ who reported enhanced nicotine reward in insulin-resistant animal models. Taken together, these findings suggest a potential link between insulin resistance and nicotine dependence, while the underlying mechanisms and directionality of this association require further investigation.

In the present study, the TyG index was higher in males than in females, indicating a sex-related difference in insulin resistance. Similar findings have been reported by Jeong et al.^17^ who observed higher TyG levels in men. This difference may reflect underlying biological and behavioral factors, including hormonal influences and sex-specific patterns of visceral adiposity, as suggested in previous studies^5^.

The mechanisms underlying the association between insulin resistance and nicotine dependence have not been fully elucidated. Previous studies have suggested several biological pathways that may be involved in this relationship. Cigarette smoking has been associated with reduced insulin sensitivity through processes such as chronic inflammation, oxidative stress, visceral adiposity, and endothelial dysfunction^17^. In addition, experimental studies have indicated that nicotine exposure may interfere with insulin signaling pathways at the cellular level^18,19^. Insulin has also been shown to modulate dopaminergic reward pathways, and impaired insulin signaling may influence reward-related responses^20,21^. In line with this, Richardson et al.^8^ reported enhanced nicotine reward in insulin-resistant animal models. Taken together, these findings provide biological plausibility for the observed association, while emphasizing the need for further research to clarify the underlying mechanisms.

### Strengths and limitations

This study adds to the limited number of human investigations evaluating the association between insulin resistance, assessed by the TyG index, and nicotine dependence, measured by the FTND. The sample size exceeded the minimum required, as determined by *a priori* power analysis, allowing for more robust estimates. Nicotine dependence was assessed using the validated FTND scale, and insulin resistance was evaluated using the TyG index, which has been increasingly applied in epidemiological research. In addition, relevant confounders, including age, sex, and cumulative smoking exposure (pack-years), were considered in the analyses, thereby strengthening the robustness of the findings.

Several limitations should be acknowledged. First, the cross-sectional design precludes any causal or directional inferences. Second, the study was conducted at a single center and included volunteers attending an outpatient clinic, which may limit generalizability and introduce volunteer bias, as participants who agreed to take part could differ systematically from those who declined (e.g. in motivation or health awareness). Third, smoking-related variables were self-reported, which may have resulted in misclassification bias. In addition, insulin resistance was estimated using the TyG index rather than direct measures. Furthermore, potential confounding factors, including body mass index, physical activity, dietary habits, and socioeconomic status, were not considered in the analyses; therefore, residual confounding cannot be excluded. These limitations should be considered when interpreting the findings.

## CONCLUSIONS

Our findings demonstrate that higher TyG index values were independently associated with greater nicotine dependence, as well as with older age and male sex. These results suggest a potential link between insulin resistance and nicotine dependence. However, given the cross-sectional design of the study, causal or directional inferences cannot be drawn. Larger, prospective studies are warranted to further clarify the direction and clinical relevance of this relationship.

## Data Availability

The data supporting this research are available from the authors on reasonable request.

## References

[1] World Health Organization. Tobacco; 2025. Accessed January 4, 2026. https://www.who.int/news-room/fact-sheets/detail/tobacco

[2] U.S. Department of Health and Human Services. The Health Consequences of Smoking—50 years of Progress: A report of the Surgeon General. U.S. Department of Health and Human Services, Centers for Disease Control and Prevention, National Center for Chronic Disease Prevention and Health Promotion, Office on Smoking and Health; 2014. Accessed January 4, 2026. https://www.ncbi.nlm.nih.gov/books/NBK179276

[3] Tiwari RK, Sharma V, Pandey RK, Shukla SS. Nicotine addiction: Neurobiology and mechanism. J Pharmacopuncture. 2020;23(1):1-7. doi:10.3831/KPI.2020.23.001 32322429 PMC7163392

[4] Heatherton TF, Kozlowski LT, Frecker RC, Fagerström KO. The Fagerström Test for nicotine dependence: A revision of the Fagerström Tolerance Questionnaire. Br J Addict. 1991;86(9):1119-1127. doi:10.1111/j.1360-0443.1991.tb01879.x 1932883

[5] Petersen MC, Shulman GI. Mechanisms of insulin action and insulin resistance. Physiol Rev. 2018;98(4):2133-2223. doi:10.1152/physrev.00063.2017 30067154 PMC6170977

[6] Kurniawan LB. Triglyceride-Glucose Index as a biomarker of insulin resistance, diabetes mellitus, metabolic syndrome, and cardiovascular disease: A review. EJIFCC. 2024;35(1):44-51. Accessed January 4, 2026. https://pmc.ncbi.nlm.nih.gov/articles/pmid/38706737/38706737 PMC11063788

[7] Kleinridders A, Pothos EN. Impact of brain insulin signaling on dopamine function, food intake, reward, and emotional behavior. Curr Nutr Rep. 2019;8(2):83-91. doi:10.1007/s13668-019-0276-z 31001792

[8] Richardson JR, Pipkin JA, O’Dell LE, Nazarian A. Insulin-resistant rats display enhanced rewarding effects of nicotine. Drug Alcohol Depend. 2014;140:205-207. doi:10.1016/j.drugalcdep.2014.03.028 24774962 PMC10292761

[9] Luo J, Rossouw J, Tong E, et al. Smoking and diabetes: Does the increased risk ever go away? Am J Epidemiol. 2013;178(6):937-945. doi:10.1093/aje/kwt071 PMC3816526 23817918

[10] Akter S, Goto A, Mizoue T. Smoking and the risk of type 2 diabetes in Japan: A systematic review and meta-analysis. J Epidemiol. 2017;27(12):553-561. doi:10.1016/j.je.2016.12.017 28716381 PMC5623034

[11] Pan A, Wang Y, Talaei M, Hu FB. Relation of smoking with total mortality and cardiovascular events among patients with diabetes mellitus: A meta-analysis and systematic review. Circulation. 2015;132(19):1795-1804. doi:10.1161/circulationaha.115.017926 26311724 PMC4643392

[12] Faul F, Erdfelder E, Buchner A, Lang AG. Statistical power analyses using G*Power 3.1: Tests for correlation and regression analyses. Behav Res Methods. 2009;41(4):1149–1160. doi:10.3758/BRM.41.4.1149 19897823

[13] IBM. IBM SPSS Statistics, Version 25.0. Accessed January 4, 2026. https://www.ibm.com/products/spss-statistics

[14] Facchini FS, Hollenbeck CB, Jeppesen J, Chen YD, Reaven GM. Insulin resistance and cigarette smoking. Lancet. 1992;339(8802):1128-1130. doi:10.1016/0140-6736(92)90730-Q 1349365

[15] Chen Z, Liu X, Kenny PJ. Central and peripheral actions of nicotine that influence blood glucose homeostasis and the development of diabetes. Pharmacol Res. 2023;194:106860. doi:10.1016/j.phrs.2023.106860 37482325 PMC13183268

[16] Cho SH, Jeong SH, Shin J, Park S, Jang SI. Short-term smoking increases the risk of insulin resistance. Sci Rep. 2022;12(1):1-9. doi:10.1038/s41598-022-07626-1 35241770 PMC8894492

[17] Jeong SH, Joo HJ, Kwon J, Park EC. Association between smoking behavior and insulin resistance using the triglyceride-glucose index among South Korean adults. J Clin Endocrinol Metab. 2021;106(11):e4531-e4541. doi:10.1210/clinem/dgab399 34160623

[18] Durlach V, Vergès B, Al-Salameh A, et al. Smoking and diabetes interplay: A comprehensive review and joint statement. Diabetes Metab. 2022;48(6):101370. doi:10.1016/j.diabet.2022.101370 35779852

[19] Li Z, Xu W, Su Y, et al. Nicotine induces insulin resistance via downregulation of Nrf2 in cardiomyocytes. Mol Cell Endocrinol. 2019;495:110507. doi:10.1016/j.mce.2019.110507 31315024

[20] Patel JC, Carr KD, Rice ME. Actions and consequences of insulin in the striatum. Biomolecules. 2023;13(3):518. doi:10.3390/biom13030518 36979453 PMC10046598

[21] Stouffer MA, Woods CA, Patel JC, et al. Insulin enhances striatal dopamine release by activating cholinergic interneurons and thereby signals reward. Nat Commun. 2015;6:8543. doi:10.1038/ncomms9543 26503322 PMC4624275

